# Magnitude and associated factors of stillbirth among women who gave birth at Hiwot Fana Specialized University Hospital, Harar, eastern Ethiopia

**DOI:** 10.18332/ejm/150354

**Published:** 2022-08-01

**Authors:** Abdusamed Mohammed-Ahmed, Aisha Abdullahi, Furo Beshir

**Affiliations:** 1 Department of Nursing and Midwifery, Harar Health Sciences College, Harar, Ethiopia; 2 Department of Medical Laboratory Science, Harar Health Sciences College, Harar, Ethiopia

**Keywords:** stillbirth, obstetrical factors, medical factors, health-service-related factors, feto-maternal factors

## Abstract

**INTRODUCTION:**

According to WHO, there are nearly 2 million stillbirths every year, one every 16 seconds. The objective of our study was to assess the frequency and associated factors of stillbirth among women who gave birth at Hiwot Fana Specialized University Hospital, Harar, eastern Ethiopia, 2021.

**METHODS:**

An institution-based retrospective cross-sectional study of medical records was conducted among 336 women who gave birth from 1 January 2020 to 31 December 2020. Maternal medical records were selected by systematic random sampling technique and a pre-tested checklist was used to collect data. Data entry and analysis were done using SPSS-version 20. Bivariate and multivariate logistic regressions were performed to identify factors associated with stillbirth. Adjusted odds ratios with 95% confidence intervals are reported.

**RESULTS:**

The frequency of stillbirth was 12.5% (95% CI: 8.1–14.6). Preterm delivery (AOR=8.10; 95% CI: 3.01–21.79), non-booking for antenatal care (AOR=2.8; 95% CI: 1.14–6.88), antepartum hemorrhage (AOR=3.16; 95% CI: 1.10–9.04), obstructed labor (AOR=2.56; 95% CI: 1.85–7.93) and eclampsia (AOR=2.84; 95% CI: 1.45–6.98) were found to be statistically significantly associated with stillbirth.

**CONCLUSIONS:**

The frequency of stillbirth in this study was high. Prematurity, non-booking for antenatal care, ante-partum hemorrhage, obstructed labor and eclampsia were independently associated for stillbirth. Therefore, we recommend that the health professionals should better work on prevention of preterm birth, active emergency obstetrical and neonatal care by boosting focused antenatal care follow-up with health education on danger signs.

## INTRODUCTION

The cut-off points for defining stillbirth varies, with the World Health Organization (WHO) defining it as a baby who dies after 28 weeks of pregnancy, but before or during birth. In United Kingdom, it is defined as a death at 24 weeks or later, whereas in the US, it is loss of a baby at or after 20 weeks of pregnancy^1,2^. Stillbirth is further classified as either early, late, or term^3^. In Ethiopia, it is defined as a fetus born dead at 28 weeks of gestation or more with a birth weight of 1000 g or more^4^.

Despite the advancement in diagnostic tools and autopsy, the cause of a large proportion of stillbirths is not known. Findings from different studies show that perinatal mortality rate is five times higher in developing than in developed regions^5,6^; 10 deaths per 1000 total births in developed regions and 50 per 1000 in developing regions. A study conducted at Babol, Northern Iran^7^, and report from WHO collaborating centers in Argentina, Egypt, India, Peru, South Africa and Vietnam, showed that the stillbirth rate was 12.5 per 1000 births^6^.

Maternal risk factors that increase the risk of fetal stillbirth include older age (>35 years), obesity and smoking^8,9^. Nulliparity, grand multiparity, obstructed labor, prolonged labor, placental abruption, placenta previa, preterm labor, premature rupture of membrane, and intrauterine growth restriction, are common obstetric factors associated with an increased risk of stillbirth, particularly in resource poor settings^8-12^.

Other maternal medical conditions such as thyroid diseases, cardiovascular disorders, asthma, kidney diseases, and diabetes, also increase the risk of stillbirth, whereas maternal infections such as malaria and syphilis also contribute to the risk of stillbirth in high burden areas, congenital anomalies of fetus, and fetal maternal hemorrhage^13-15^.

Globally two-thirds to three-quarters of stillbirths may occur during the antenatal period before labor begins, which are often caused by insults that occur *in utero* during the antenatal period. Some of such causes include bacterial infection, birth defects especially pulmonary hypoplasia, maternal diabetes, hypertensive diseases in pregnancy, maternal alcohol consumption, cigarette smoking, post-term pregnancy, abruption placentae, radiation poison, physical trauma, rhesus disease, umbilical cord accidents and intra uterine growth restriction. Other associated factors include advanced maternal age, low socioeconomic status, poor maternal education, nulliparity or grand multiparity, and previous stillbirth^16-18^.

Intrapartum stillbirths are usually the result of fetal distress and/or obstructed labor, which often reflect poor quality of clinical care during labor and delivery. The potential contribution of antenatal care and good partographic monitoring during labor will largely help in detecting these risk factors and prevent stillbirth^19,20^. According to research done in South Africa, there is no significant effect of gestational age at first ANC visit on stillbirth whereas research conducted in Southern Nigeria shows that the later the gestational age at first ANC, the higher the fetal mortality^21^.

Obstetric and medical factors complicate pregnancy and hence endanger the life of the fetus during pregnancy. Among these factors, hypertensive disorders of pregnancy, including preeclampsia, gestational hypertension, eclampsia, and/or superimposed preeclampsia/eclampsia were the most common risk factors which have been associated with stillbirth in many studies, and diagnosed in 8% of pregnancies but may affect as many as 20% of pregnancies. A study done in Pakistan^22^ revealed that a woman with hypertensive disorder of pregnancy is at much higher risk of developing stillbirth than non-hypertensive woman. Also stillbirths in the first two pregnancies have common biological causes beyond any known risk factors of stillbirth that may develop during the second pregnancy. Women who had previously had a stillbirth, were more likely to have another stillbirth (4.6% vs 1.4%) even though evidence regarding the recurrence of stillbirth remains controversial^21-25^.

There is limited evidence of the frequency of stillbirth and its associated factors among women who give birth in low-income countries like Ethiopia. Thus, the aim of this study was to assess the magnitude of stillbirth and factors associated with it among women who gave birth at Hiwot Fana Specialized University Hospital, Harar, eastern Ethiopia.

## METHODS

### Study design and setting

An institution-based cross-sectional study design was conducted at Hiwot Fana Specialized University Hospital, Harar, Ethiopia, from 15 May to 31 May 2021. This is one of the federal tertiary referral teaching hospitals directly run under Haramaya University, and it provides service to about 232000 clients annually including patients referred from nearby regions and zones. On average, 350 patients visit the hospital outpatient and emergency units, daily. The hospital gives service under different clinical disciplines including obstetrics and gynecology. Nearly 1656 attended antenatal care and around 5074 deliveries were performed in 2020. Around 754 women receive abortion care in the hospital, annually^26^.

### Population

All records of women who gave birth at Hiwot Fana Specialized University Hospital from 1 January to 30 December 2020 were included in the study. Exclusion criteria included records that were incomplete or lacking important research variables, missing records (unable to be found), or lost records, were excluded from the study.

### Sample size determination

Sample size was calculated by using a single population proportion formula by taking the proportion (*p*=8.6%) of stillbirths at Negest Elene Mohammed Memorial General Hospital in Hosanna Town^27^ with an assumption of 5% margin of error and 95% CI; where *p* is the proportion of stillbirths, n is the minimum sample size, *d* is degree of precision (tolerated error) (5%), and z_α/2_ is the 95% confidence interval (which is 1.96). This gave a final sample size of 336. A systematic random sampling technique was used to select maternal records to be reviewed. The list of all registration cards of mothers who gave birth from 1 January to 30 December 2020 was used as sampling frame. Sampling interval was determined by dividing the total number (N) of mothers who gave birth during the selected year by the final sample size (n), by using the formula: K=N/n=14.29. By selecting the first record by simple random sampling technique, the mothers’ records were selected using a delivery registration number at every 14th interval until the final sample size was reached.

### Data collection instruments

The data collection tool was adapted from various literature^27-32^. It possesses five parts: 1) three questions on sociodemographic characteristics, 2) seventeen questions on obstetric factors, 3) six questions on maternal medical factors, 4) two questions on health-related factors, and 5) five questions on fetal factors.

### Data collection procedure

The data were collected by retrieving medical record numbers using checklists that were prepared in English. The data collections were conducted and supervised by three BSc midwives and 2 MSc midwives, respectively. One-day training was given to the data collectors and supervisors on how they should approach the study and fill in the checklist.

### Ethical considerations

Ethical clearance was obtained from health research ethical and technical review committee of Harar Health Science College. Official letter of cooperation, which was written by the Research and Development Vice Dean of the College and given to the administrators of Hiwot Fana Specialized University Hospital. The supportive letter that was obtained from the hospital was given to the concerned body to have official communication before conducting the data collection. Privacy and confidentiality of the data was ensured.

### Definitions

Stillbirth is defined as a fetus born dead at 28 weeks of gestation or more with a birth weight of 1000 g or more^33^. Fresh stillbirth is an intrauterine death of a fetus during labor or delivery where the fetus showed no signs of degenerative changes. A macerated stillbirth is defined as the intrauterine death of a fetus sometime before the onset of labor, where the fetus showed signs of degenerative changes^34^. Unbooked woman is defined as a pregnant woman who has not attended any antenatal clinic session throughout the pregnancy with a skilled attendant (trained medical personnel) before presentation in labor, whereas a booked pregnant woman is one who attended at least one antenatal clinic session during pregnancy by trained personnel^35^.

### Data processing and analysis

After data collection, the questionnaire was checked for completeness and coded before data entry. The data were cleaned, entered and analyzed using SPSS version 20. Bivariate analysis was run to check the association of independent variables with the dependent variable. Variables showing p<0.2 in binary logistic regression were entered into multivariate logistic regression analysis to identify predicting factors of stillbirth by controlling for confounding variables. Those variables with p<0.05 at alpha 5% were considered as statistically significant variables for stillbirths. Odds ratios with 95% confidence interval were also used to measure the degree of association between independent variables and the dependent variable.

## RESULTS

### Participant characteristics

In this study, 336 records of mothers were assessed. Among these, 176 (52.4%) were aged 18–24 years; 167 (49.7%) respondents were rural residents and 284 (85.5%) were married. A total of 112 (33.3%) mothers had a history of anemia, 5 (1.5%) had a history of pregnancy related diabetes mellitus, and 6 (1.8%) had a history of HIV (Supplementary file Tables 1 and 2).

### Obstetric characteristics

Among 336 reviewed maternal records, 127 (37.8%) and 162 (48.2%) records were primiparous and multiparous, respectively. Thirty-nine (11.6%) had a history of abortion whereas 42 (12.5%) had a history of stillbirth. Regarding hypertensive disorder of pregnancy, 21 (6.3%) of the mothers had a history of preeclampsia whereas 49 (11%) had previously experienced eclampsia. Concerning their gestation, 30 (8.9%) of mothers had a pre-term delivery while 5 (1.5%) delivered post-term. On the course of the labor process, 8 (2.4%) and 5 (1.5%) of the mothers experienced obstructed labor and cord prolapse, respectively (Table 1). Out of 336 reviewed maternal records, 42 [12.5% (95% CI: 8.1–14.6)] had a stillbirth.

**Table 1:** Obstetric characteristics and maternal outcomes among mothers who gave birth at Hiwot Fana Specialized University Hospital, Harar, eastern Ethiopia, 2021 (N=336)

*Characteristics*	*Stillbirth*		*Total n (%)*
	*Yes n (%)*	*No n (%)*	
**Parity**			
Primiparous	18 (5.4)	109 (32.4)	127 (37.8)
Multiparous	18 (5.4)	144 (42.8)	162 (48.2)
Grand multiparous	6 (1.8)	41 (12.2)	47 (14.0)
**Obstructed labor**			
Yes	1 (0.3)	7 (2.1)	8 (2.4)
No	41 (12.2)	287 (85.4)	328 (97.6)
**Premature rupture of membrane**			
Yes	9 (2.7)	12 (3.6)	21 (6.3)
No	33 (9.8)	282 (83.9)	315 (93.8)
**Type of antepartum hemorrhage**			
Abruption	14 (46.7)	4 (13.3)	18 (60)
Previa	5 (16.7)	7 (23.3)	12 (40)
**Cord prolapse**			
Yes	1 (0.3)	4 (1.2)	5 (1.5)
No	41 (12.2)	290 (86.3)	331 (98.5)
**History of abortion**			
Yes	12 (3.6)	27 (8.0)	39 (11.6)
No	30 (8.9)	267 (79.5)	297 (88.4)
**History of stillbirth**			
Yes	12 (3.6)	30 (8.9)	42 (12.5)
No	27 (8.0)	267 (79.5)	294 (87.5)
**Onset of labor**			
Spontaneous	38 (11.3)	268 (79.8)	306 (91.1)
Induced	4 (1.2)	26 (7.7)	30 (8.9)
**Labor started before admission**			
Yes	12 (3.6)	51 (15.2)	63 (18.8)
No	30 (8.9)	243 (72.3)	273 (81.3)
**Duration of labor before admission** (hours)			
<24	26 (41.3)	18 (28.6)	44 (69.9)
≥24	6 (9.5)	13 (20.6)	19 (30.1)
**Chorioamnionitis**			
Yes	1 (0.3)	7 (2.1)	8 (2.4)
No	1 (12.2)	287 (78.4)	328 (97.6)
**Gestational age**			
Pre-term	12 (3.6)	18 (5.4)	30 (8.9)
Term	30 (8.9)	271 (80.7)	301 (89.6)
Post-term	0 (0)	5 (1.5)	5 (1.5)
**Preeclampsia**			
Yes	9 (2.7)	12 (3.6)	21 (6.3)
No	33 (9.8)	282 (83.9)	315 (93.8)
**Eclampsia**			
Yes	32 (9.5)	17 (5.1)	49 (14.6)
No	10 (2.9)	277 (82.4)	287 (85.4)
**Mode of delivery**			
Spontaneous vaginal delivery	30 (8.9)	251 (74.7)	281 (83.6)
Other*	12 (3.6)	43 (12.8)	55 (16.4)
**Total**	42 (12.5)	294 (87.5)	336 (100)

* Cesarean section or instrument-assisted vaginal delivery.

### Health service-related factors

A total of 252 (75%) mothers had an ANC visit, and 84 (25.0%) came to hospital with a referral (Table 2).

**Table 2:** Health service-related factors of mothers who gave birth at Hiwot Fana Specialized University Hospital, Harar, eastern Ethiopia, 2021 (N=336)

*Variables*	*Stillbirth*		*Total n (%)*
	*Yes n (%)*	*No n (%)*	
**Antenatal care visits**			
Yes	11 (3.3)	241 (71.7)	252 (75)
No	31 (9.2)	53 (15.6)	84 (24.8)
**Reason for coming to hospital**			
Referral	10 (2.9)	74 (22.0)	84 (25.0)
Without referral	32 (9.5)	220 (65.5)	252 (75.0)
Total	42 (12.5)	294 (87.5)	336 (100)

### Fetal-related factors of respondents

Out of 336 mothers, 6 (1.8%) gave birth to a congenitally malformed baby, 70 (20.8%) had a low birthweight baby, and 8 (2.4%) had an intrauterine growth retardation stillbirth. A total of 27 (8.0%) mothers delivered an intrauterine growth retarded (IUGR) fetus (Table 3).

**Table 3:** Fetal related factors of mothers who gave birth at Hiwot Fana Specialized University Hospital, Harar, eastern Ethiopia, 2021 (N=336)

*Variables*	*Stillbirth*		*Total n (%)*
	*Yes n (%)*	*No n (%)*	
**Congenital anomaly**			
Yes	3 (0.9)	3 (0.9)	6 (1.8)
No	39 (11.6)	291 (86.6)	330 (98.2)
**Fetal weight at birth** (g)			
<2500	5 (1.5)	65 (19.3)	70 (20.8)
≥2500	37 (11.0)	229 (68.2)	266 (79.2)
**Intrauterine growth retardation**			
Yes	8 (2.4)	19 (5.7)	27 (8.0)
No	34 (10.1)	275 (81.8)	309 (92.0)
**Malpresentation and malposition**			
Yes	8 (2.4)	16 (4.8)	24 (7.1)
No	34 (10.1)	278 (82.7)	312 (92.9)
**Sex of the fetus**			
Male	28 (8.3)	188 (55.9)	216 (64.3)
Female	14 (4.2)	106 (31.5)	210 (35.7)
**Total**	42 (12.5)	294 (87.5)	336 (100)

### Factors associated with stillbirth

In bivariate analysis with p<0.2, the factors found to be statistically significantly associated with stillbirth were maternal age, lack of antenatal care (ANC) visit, anemia, history of abortion, history of stillbirth, chorioamnionitis, birth weight, gestational age at birth, premature rupture of membrane (PROM), antepartum hemorrhage (APH), obstructed labor, and eclampsia, at the 95% confidence interval. Among the above variables, gestational age, lack of ANC visit, history of stillbirth, APH, obstructed labor and eclampsia were found to be statistically significantly associated with stillbirth in multivariate logistic regression at alpha 5%.

Mothers who had no ANC visit were two times more likely to have stillbirth than those who had an ANC visit (AOR=2.8; 95% CI: 1.14–6.88), whereas mothers who had APH were three times more likely to develop stillbirth compared to women who did not (AOR=3.16; 95% CI: 1.10–9.04). Also, mothers who developed obstructed labor were two times (AOR=2.56; 95% CI: 1.85–7.93) more likely to have a stillbirth than mothers who gave birth free of obstructed labor, while those who had a history of eclampsia were three times more likely to have a stillbirth compared to women who did not (AOR=2.84; 95% CI: 1.45–6.98) (Table 4).

**Table 4:** Bivariate and multivariate logistic regression analysis of factors associated with stillbirth among mothers who gave birth at Hiwot Fana Specialized University Hospital, Harar, eastern Ethiopia, 2021 (N=336)

*Variables*	*Stillbirth*		*OR (95% CI)*	*AOR (95% CI)*
	*Yes n (%)*	*No n (%)*		
**Maternal age** (years)				
18–24	19 (5.7)	157 (46.7)	2.03 (0.92–44.48)	1.24 (0.41–3.68)
25–34	20 (5.9)	109 (32.4)	0.66 (0.19–2.24)	1.58 (0.25–9.87)
>35 (Ref.)	3 (0.9)	28 (8.3)	1	1
**Anemia**				
Yes	31 (9.2)	81 (24.1)	0.23 (0.21–0.45)	1.44 (0.57–3.62)
No (Ref.)	11 (3.3)	213 (63.4)	1	1
**History of abortion**				
Yes	12 (3.6)	27 (8.0)	2.08 (0.92–4.48)	0.75 (0.24–2.32)
No (Ref.)	30 (8.9)	267 (79.5)	1	1
**History of stillbirth**				
Yes	12 (3.6)	30 (8.9)	2.62 (1.11–4.67)	1.67 (1.16–6.63)
No (Ref.)	27 (8.0)	267 (79.5)	1	1
**Gestational age**				
Term (Ref.)	30 (8.9)	271 (80.7)	1	1
Preterm***	12 (3.6)	18 (5.4)	5.05 (2.53–10.09)	8.10 (3.01–21.79)
Post-term	0 (0)	5 (1.5)		
**Premature rupture of membrane**				
Yes	9 (2.7)	12 (3.6)	2.46 (11.26–4.80)	1.71 (0.65–4.49)
No (Ref.)	33 (9.8)	282 (83.9)	1	1
**Antepartum hemorrhage**				
Yes*	12 (3.6)	18 (5.4)	4.93 (2.34–10.37)	3.16 (1.10–9.04)
No (Ref.)	30 (8.9)	276 (82.1)	1	1
**Obstructed labor**				
Yes**	1 (0.3)	7 (2.1)	2.26 (1.60–5.61)	2.56 (1.85–7.93)
No (Ref.)	41 (12.2)	287 (85.4)	1	1
**Eclampsia**				
Yes**	32 (9.5)	17 (5.1)	5.03 (2.69–10.45)	2.84 (1.45–6.98)
No (Ref.)	10 (2.9)	277 (82.4)	1	1
**ANC follow-up**				
Yes**	11 (3.3)	241 (71.7)	4.11 (1.99–8.46)	2.80 (1.14–6.88)
No (Ref.)	31 (9.2)	53 (15.6)	1	1

AOR: adjusted odds ratio. Significant at: *p=0.03, **p=0.02, ***p=0.001.

## DISCUSSION

Stillbirth rate is a key indicator of women’s health and quality of care in pregnancy and child birth. Also, it is a marker of utilization and adequacy of obstetric care; and an important source of medical litigation in some countries. Globally, at least 2.65 million stillbirths occur every year which accounts for over 7178 deaths per day. The majority (98%) of stillbirths occur in low- and middle-income countries, and more than half (55%) of these happen in rural Sub-Saharan Africa. Ten populous countries (India, Pakistan, Nigeria, China, Bangladesh, Democratic Republic of the Congo, Ethiopia, Indonesia, Tanzania, and Afghanistan) account for two-thirds of all third-trimester stillbirths^15,36,37^.

Although some developed countries report a stillbirth rate of 3 per 1000 births, a ten-fold increase is noted in some settings in Sub-Saharan Africa and South-East Asia with reported stillbirth rates of ≥30 per 1000 births. Every stillbirth is a tragedy and life loss. There are in addition many psychosocial consequences^38-40^. Advances in prenatal, intrapartum and neonatal care in developed countries significantly have reduced perinatal mortality, however, in developing regions, poor quality or low access to healthcare results in 50–88% of overall perinatal mortality in the various regions of the world and it is a close reflection of the perinatal mortality rate of the community^41,42^.

This study revealed that the prevalence of stillbirth was 12.5% (95% CI: 8.1–14.6), higher than the study conducted in Ethiopia, Aksum (3.68%), Yirgalem Hospital (9.2%), Nigerian referral hospitals (3.96%) and India (4.0%), Pakistan (1.6%), overall in Pakistan (5.69%), and Babol, Northern Iran (10.51%)^6,14,28,43-45^. This difference might be due to the study design, sociodemographic characteristics of study participants, and/or the technological differences.

In this study, women who did not attend antenatal care were more likely to have a stillbirth than their counterpart. This finding was supported by study done at Felegehiwot referral hospital, Ethiopia^46^, and the study conducted at Aksum General Hospital^28^, which reveals the odds of developing stillbirth is high among women who do not attend ANC follow-up. The association might be due to antenatal visits of the pregnant mothers, which are very important as they provide chances for monitoring the fetal wellbeing and allow timely intervention for feto-maternal protection. The finding of this study is higher than that for the same factor of the study conducted at Gondar, Ethiopia^47^. The possible explanation for this difference could be variation in study area and sociodemographic characteristics of study participants.

Mothers who had APH were three times more likely to have a stillbirth compared to women who did not. This study was similar to the study done in Gondar, Ethiopia^47^ and Pakistan^9^. This may be due to the fact that APH during pregnancy may result from a placental abnormality which results in excess bleeding and anemia, and decreased placental perfusion which in turn results in intrauterine hypoxia, development of infection and premature delivery that increases the risk of stillbirth.

According to this study mothers who had obstructed labor were two times more likely to have a stillbirth compared to women who did not. This study was similar to the study conducted in Pakistan^9^. This may be due to delayed presentation of the mothers at a health facility, poor referral system, delayed diagnosis and poor emergency preparedness and response, resulting in rupture of the uterus which increases stillbirth.

Mothers who had history of eclampsia were almost three times more likely to have stillbirth compared to women who did not. This is in accordance with studies done in Pakistan and Iran^6,9^. This variation could be due to hypertensive disorders of pregnancy resulting in complicated labor and intrauterine growth restriction which lead to small for gestational age and preterm labor, resulting in increased chances of stillbirth.

### Limitations

As this study is facility-based, the reported stillbirth rates and determinants exclude stillbirths that occur in births outside facilities. Therefore, the findings do not necessarily reflect the essential predisposing factors that lead to stillbirths. Furthermore, additional personal and environmental factors may have influenced the stillbirth, which we were not able to document. Finally, the results of this study may not be generalizable to other populations, however, they provide regional evidence of the magnitude of stillbirths in Ethiopia.

## CONCLUSIONS

The prevalence of stillbirths found in this study was 12.4%. Factors such as non-booking for antenatal care, antepartum hemorrhage, obstructed labor and eclampsia were predictors of stillbirth. The factors identified in this study can be prevented and managed by providing appropriate care during the ante-partum and intra-partum periods. Encouraging ANC service utilization, awareness creation on obstetrical danger signs and pregnancy complications, facilitating a smooth referral system and/or following feto-maternal conditions by partographs in order to prevent obstructed labor is recommended.

## Data Availability

The data supporting this research are available from the authors on reasonable request.
